# Prevalence of cardiovascular medication on secondary prevention after myocardial infarction in China between 1995-2015: A systematic review and meta-analysis

**DOI:** 10.1371/journal.pone.0175947

**Published:** 2017-04-20

**Authors:** Min Zhao, Kerstin Klipstein-Grobusch, Xin Wang, Johannes B. Reitsma, Dong Zhao, Diederick E. Grobbee, Ian Graham, Ilonca Vaartjes

**Affiliations:** 1 Julius Global Health, Julius Center for Health Sciences and Primary Care, University Medical Center Utrecht, Utrecht, Utrecht, The Netherlands; 2 Division of Epidemiology & Biostatistics, School of Public Health, Faculty of Health Sciences, University of the Witwatersrand, Johannesburg, Gauteng, South Africa; 3 Julius Center for Health Sciences and Primary Care, University Medical Center Utrecht, Utrecht, Utrecht, The Netherlands; 4 Capital Medical University Beijing Anzhen Hospital, Beijing, Beijing, China; 5 Global Geo and Health Data Centre, Utrecht University, Utrecht, Utrecht, The Netherlands; 6 Trinity College Dublin, Dublin, Dublin, Ireland; University of Bologna, ITALY

## Abstract

**Background:**

Myocardial Infarction (MI) has become a major cause of morbidity and mortality in China, but little is known about the prevalence of guideline-recommended cardiovascular medications after MI events over the last two decades. This systematic review and meta-analysis aims to summarize cardiovascular medication use between 1995–2015 and to assess factors in associated with the trends in cardiovascular medications.

**Method:**

A systematic search was conducted in four databases (Pubmed, Embase, CENTRAL, and CNKI) to obtain observational studies published between 1995 and 2015, reporting on the use of cardiovascular medications in China. Risk of bias of individual studies was appraised and selected studies were pooled for estimated prevalence of cardiovascular medication. Prevalence of cardiovascular medication use for 1995 and 2015 was estimated by random effects meta-regression model.

**Results:**

From 13,940 identified publications, 35 studies, comprising 28,000 patients, were included. The pooled prevalence for aspirin, beta-blockers, statins, ACE-Inhibitors, ACE-Inhibitor/ARBs and nitrates was 92% [95% confidence interval (CI): 0.89–0.95], 63% (95% CI: 0.57–0.69), 72% (95% CI: 0.60–0.82), 49% (95% CI: 0.41–0.57), 59% (95% CI: 0.48–0.69) and 79% (95% CI: 0.74–0.91), respectively. A significant increase in beta-blocker and statin use and a decrease of nitrate use was observed over time. The estimated prevalence of beta-blockers, statins, and nitrates was 78%, 91.1%, and 59.3% in 2015, compared to 32%, 17% and 96% in 1995, respectively.

**Conclusion:**

Cardiovascular medication use after MI is far from optimal in Chinese patients, even though the prevalence of use increased over the period 1995–2015. With a rapidly increasing number of MI patients in China, a comprehensive strategy on secondary prevention is warranted.

**Systematic review registration:**

PROSPERO (CRD42015025246)

## Introduction

Rapidly increasing per capita income and an aging population have led to profound demographic and epidemiologic changes in China. [1–3] Cardiovascular disease (CVD) has become the leading non-communicable disease over the past two decades.[1] The number of ischemic heart disease events in China significantly increased from 0.75 million in 1990 to 1.4 million in 2013 [2]; Currently, one million deaths are caused by myocardial infarction (MI) annually[1,3].

Reflecting this, healthcare system reforms, improved medical insurance coverage and evidence-based guideline recommendations have been recently introduced by the Chinese government. This has led to some remarkable strides in MI management with better quality of care and more effective medical therapy.[1,3–5] Widespread and long-term medical therapy by using cardiovascular medications for secondary prevention after MI events have been highly recommended in the Chinese prevention guideline to reduce mortality rates from MI and recurrent acute cardiac events.[6] However, the prevalence of guideline-recommended cardiovascular medication in daily practice has been rarely assessed. There is little solid evidence about the current use and changes of cardiovascular medications after a MI event in secondary prevention, especially for patients after hospital admission.[3] It is of concern whether cardiovascular medication has been properly implemented in daily practice.

Therefore, we aimed to perform a comprehensive review and meta-analysis of the observed cardiovascular medication use in Chinese MI patients after their hospital admissions. The specific aims of our study were: i) to summarize the pooled prevalence of five specific classes of cardiovascular medication use in patients with previous MI in China from 1995 to 2015; and ii) to identify whether specific factors, such as study characteristics are associated with the use of cardiovascular medications.

## Materials and methods

### Search strategy and eligibility criteria

This review was written in accordance with the guidelines issued by PRISMA for reporting systematic reviews and meta-analysis (S1 Checklist)[7,8] and registered in the registry for systematic reviews PROSPERO (registration number: CRD42015025246)[9]. A systematic literature search of observational studies, published between January 1, 1995 and August 10, 2015, was performed in the following databases: Pubmed/MEDLINE, EMBASE, Cochrane Central Register of Controlled Trials (CENTRAL), and China National Knowledge Infrastructure (CNKI). CNKI is an electronic platform created to integrate significant Chinese knowledge-based information resources. A combined text and subject heading terms (Mesh and EMTree) related to the observed use of cardiovascular medication (aspirin, beta-blockers, ACE-inhibitors <ACE-I>, statin, and nitrates) among adults in China were used (S1 Table).

Articles were excluded from the review if: i) published in a language other than English or Chinese; ii) focused on primary care of MI only; iii) reported medication use for CVD but not specified for MI; iv) focused on cardiovascular medication use before or during hospital admission; v) performed outside of China or conducted in non-Chinese populations; vi) performed as randomized control trials on cardiovascular medication evaluation; vii) animal studies, study protocols, bimolecular studies, case reports, non-peer reviewed published reports of proceedings, and reviews (S1 Table).

In the current review, studies reporting broadly on Acute Coronary Syndromes (ACS) were included. Apart from explicit clinical diagnosis, current guidelines and evidence indicate no difference for medical treatment and prevention level for both ACS and MI. [10–12] Furthermore, angiotensin receptor blockers (ARB) are clinical recommended when patients do not tolerate ACE-Is.[6,11,12] Therefore, studies reporting ACE-I/ARB were also included. ACE-I/ARB was considered as an independent medication category and hence analyzed separately.

### Selection process

Search results were downloaded into Refwork for Pubmed, EMBASE, and CENTRAL hits and EndnoteX7 for CNKI. Two independent reviewers (MZ and XW) screened all articles by title and abstract for inclusion and exclusion criteria. Duplicate records were automatically removed by reference management software. Any disagreements between the two reviewers on paper selection were discussed by explicit selection rules, and the full-text reviewed if necessary. For eligible articles, the full text of eligible articles were retrieved and assessed by two reviewers (MZ and XW) following processes.

### Data extraction

A standardized data extraction form was designed to capture study characteristics, participants’ characteristics, and outcome measures. Extracted items included were: sample size, performed geographic area, year of survey, participation rate, mean age, proportion of women, known history (coronary heart disease, MI, hypertension, dyslipidaemia, and diabetes), and prevalence of cardiovascular medication (aspirin, beta-blockers, statins, ACE-Is, ACE-I/ARB, and nitrates) in each study. If multiple publications were derived from one study, all unique data were extracted and combined directly into a single data extraction form. If the reported study characteristics differed from publication to publication in the same study, the publication with most explicit participants’ characteristics and outcome measures was extracted and others were excluded. When results were published multiple times, the data was used only once. Extraction was done by a single reviewer. Lack of clarity during the extraction process was resolved by consulting the second reviewer (XW).

### Quality assessment

To appraise the risk of bias of individual studies we used a tool developed by Li et al (S2 Table)[13]. The tool consists of five items that assess the quality of the study design, study population, participation rate, participants’ characteristics, and outcome. Presence of bias was assessed by scoring (low risk = 2, moderate risk = 1, high risk = 0) each of the five items. Studies with a summative score below 6 were excluded from this review (S2 Table).

### Data analysis

The prevalence of cardioprotective medication use was defined as the number of MI patients using the medication of interest divided by the total number of MI patients and displayed as proportions. Heterogeneity was presented among included studies as the I^2^ statistics>75% and P value of Q statisitcs<0.05. Hence, a random-effects model to meta-analyse the logit-transformed proportions to obtain a pooled estimate together with a 95% confidence interval (CI) was used. The model took into account the precision by which this proportion has been estimated in each study using the binomial distribution and incorporating any additional variability beyond chance that exists between studies.

To identifly factors associated with the use of cardiovascular medication, we added several study characteristics as covariates to our random effect meta-regression models. The following characteristics extracted from individual studies were examined independently: year of survey, mean age, proportion of women and geographic area. The meta-regression models, showing statistically significant association between specific study characteristics and cardioprotective medication use, were then used to estimate the prevalence of cardiovascular medication use for 1995 and 2015. All tests were two tailed with statistical significance at 0.05 level.

Statistical analyses were performed by using R ‘metafor’ package.[14]

## Results

### Study selection

**Fig 1:**
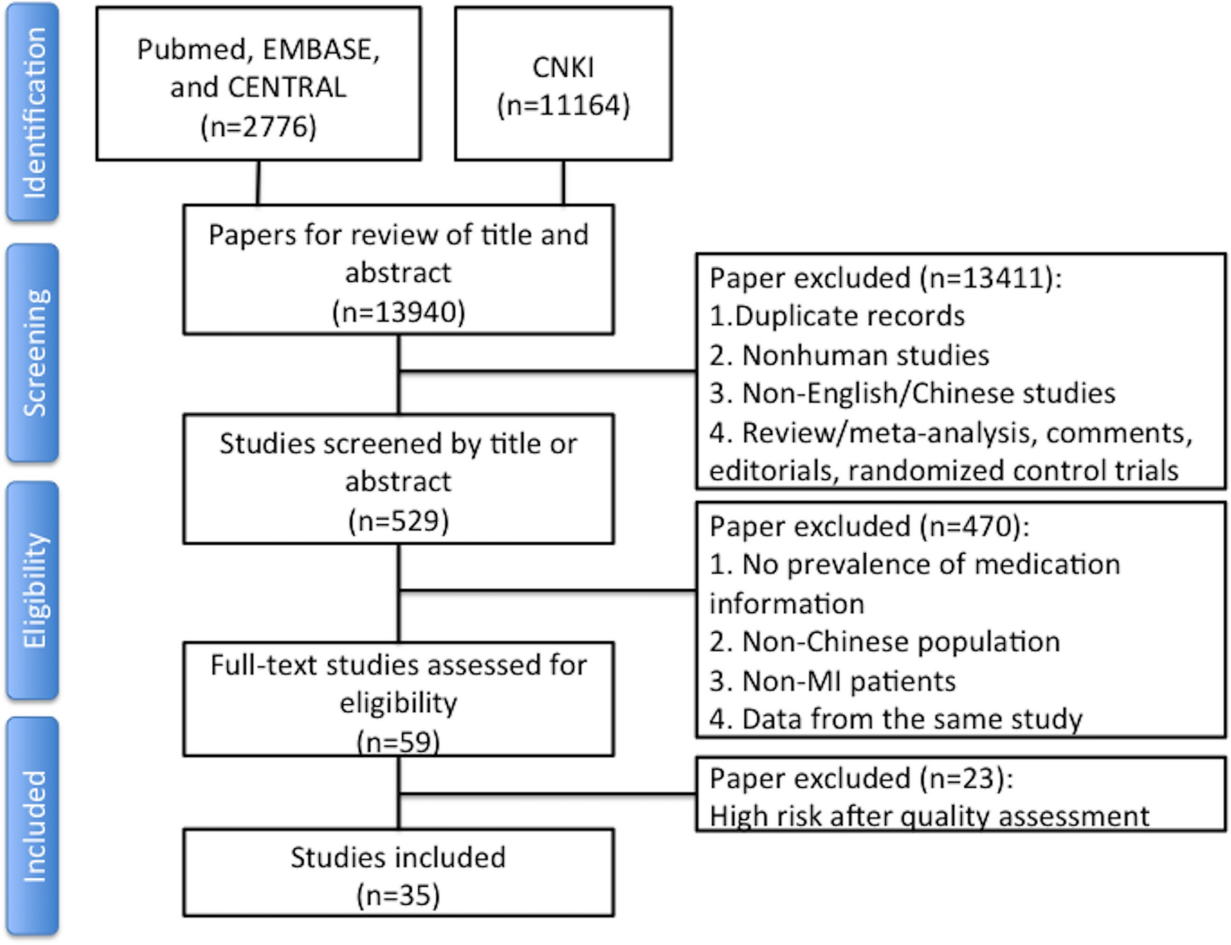
Flowchart of records screened and included in the systematic review. Graphical representation of the systematic search. Abbreviations in the flowchart: CENTRAL: Cochrane Register of Controlled Trials; CNKI: China National Knowledge Infrastructure.

The initial search resulted in 13,940 potentially relevant articles, of which 13,411 were excluded by screening title/abstract and 490 by full-text review (Fig 1). The main reasons for exclusion included: non-observational studies; non-English/Chinese studies; non-Chinese participants; non-MI participants; no data on the use of cardiovascular medication. Details are provided in the flow chart (Fig 1). After risk of bias assessment, 35 articles were selected, of which three were written in English. Detailed information on the basis assessment for the individual studies is provided in S2 Table.

### Study characteristics

Table 1 summarizes the key characteristics of the included articles and outcomes of interest. Of the 35 studies, 25 included prevalence information on aspirin use [15–38], 30 on beta-blocker use [15–21,24–35,37–46], 24 on statin use [16–21,24–33,37,38,40,42,44,45,47,48], 11 on ACE-I use [15–17,21,26,39,41–43,49], 18 on ACE-I/ARB use [17–19, 24, 27, 29, 32, 35, 37, 38, 40, 43, 46, 48], and 12 on nitrate use [15,17,19,24,27,32,38–41,43,48].

**Table 1:** Key characteristics of the 35 selected studies.

Study (year)	Language	Survey Year	Area	Sample size	Mean age	Women (%)	Discharge Med^a^	Prevalence (%)							
								Aspirin	BB	Statin	ACE-I	ACE-I /ARB		Nitrate	
Liu (1999) [39]	CHN	1997	Nationwide	400	60.9	34.3	N	NR	44.4	NR	38.5	NR	85.5	
Fang (2003)[49]	CHN	2002	North	122	68.0	42.6	Y	NR	NR	NR	47.1	NR	NR	
Zhao (2004)[40]	CHN	2002	South	226	66.7	27.9	N	NR	37.5	49.6	NR	NR	95.6	
Wu (2005)[47]	CHN	1999	North	227	61.2	22.0	N	NR	N/A	40.4	NR	NR	NR	
Wang (2005)[15]	CHN	2002	Central	178	63.2	11.8	N	90.7	27.0	NR	31.5	NR	87.6	
OASIS (2005)[41]	CHN	2001	Nationwide	2294	62.8	37.8	Y	NR	67.5	NR	59.1	NR	96.6	
Xiang (2006)[16]	CHN	2004	South west	119	68.0	29.4	N	84.9	35.3	15.1	43.7	NR	NR	
Fang (2006)[42]	CHN	2004	North	247	69.0	31.2	N	NR	40.9	14.6	47.7	NR	NR	
Yang (2006)[43]	CHN	2005	Central	174	61.2	24.1	N	NR	27.6	NR	51.7	NR	86.8	
Peng (2008)[17]	CHN	2007	East	143	68.9	22.2	N, 1 mth	96.9	74.8	76.3	55	NR	NR	
Chai (2008)[18]	CHN	2006	North	344	60.1	22.7	Y	99.4	77.3	98.0	NR	76.7	NR	
Gui (2008)[19]	CHN	2007	East	209	72.2	19.6	Y	93.6	59.2	49.2	NR	60.3	96.8	
Ni (2009)[20]	CHN	2007	East	432	66.5	19.4	N	94.4	87.7	87.9	NR	NR	NR	
Bi (2009)[21]	ENG	2006	Nationwide	2901	64.5	32.7	Y	92.7	70.0	80.4	67.8	NR	NR	
Zhou (2010)[22]	CHN	2008	South	220	58.4	36.8	Y	88.5	NR	NR	NR	NR	NR	
Wang (2010)[23]	CHN	2009	East	192	57.5	32.8	Y	90.6	NR	NR	NR	NR	NR	
Zhao (2010)[24]	CHN	2005	South	522	65.7	27.2	N	81.9	59.6	57.8	NR	69.3	77.6	
Yan (2010)[25]	ENG	2008	North	422	59.2	21.8	N	79.3	54.3	55.8	NR	35.3	NR	
Yao (2011)[26]	CHN	2009	North	200	66.5	38.0	N,1 yr	96.5	72.5	84.5	NR	NR	NR	
Liu (2011)[27]	CHN	2010	East	206	66.0	30.6	N,6 mths	86.6	56.3	69.7	NR	42.3	72.5	
Zhou (2011)[44]	CHN	2009	North	723	NA	NA	N, 3 yrs	87.8	68.6	67.3	45.3	NR	NR	
Zhang (2011)[28]	CHN	2010	North east	156	64.3	24.7	N	92.9	67.3	82.1	NR	52.6	NR	
Han (2011)[29]	CHN	2009	East	249	66.4	30.9	N,6 mths	99.1	93.6	99.1	NR	95.0	NR	
Zhang (2012)[45]	CHN	2009	Nationwide	10753	NA	NA	N	89.2	61.9	59.3	NR	47.2	NR	
Xu (2012)[30]	CHN	2010	North	180	69.0	37.2	N	91.7	66.7	81.1	NR	58.9	NR	
Han (2012)[31]	CHN	2011	North	563	NA	42.6	N	81.6	49.1	56.0	NR	NR	NR	
Li (2013)[32]	CHN	2014	East	1319	67.0	24.2	N	98.2	61.2	92.5	NR	62.2	47.3	
Wang (2013)[46]	CHN	2009	Nationwide	221	NA	28.5	N	NR	53.4	NR	NR	66.5	NR	
Yang (2013)[33]	ENG	2010	North	808	60.2	22.2	N	79.5	65.0	60.5	NR	49.4	NR	
Zhang (2014)[34]	CHN	2012	North east	218	NA	26.1	N, 2yrs	98.6	67.4	NR	NR	65.6	NR	
Li (2014)[35]	CHN	2013	North	180	63.7	38.9	Y	92	72.0	93.0	NR	74.0	NR	
Yang (2014)[36]	CHN	2013	Central	134	53.7	32.1	N, 1mth	65.7	NR	NR	NR	NR	NR	
Tian (2014)[37]	CHN	2013	North	490	64.3	34.3	N, 6mths	89.8	78.8	72.2	NR	48.2	NR	
Xiao (2014)[48]	CHN	2011	North east	110	66.5	40.9	N	NR	49.1	NR	NR	54.5	66.4	
Zhang (2015)[38]	ENG	2008	North	2514	60.4	14.0	N	77	69.0	28.0	NR	11.0	NR	

CHN: Chinese; ENG: English; Discharge med: discharge medications; mths: months; yrs: years; BB: beta-blockers; ACE-I: ACE-inhibitor; ARB: angiotensin receptor blocker; NA: not applicable; NR: not recorded in study; Y: yes; N: No.

^a^ Discharge medication is referring discharge medications after hospital admission; ‘Y’: the recorded prevalence of cardiovascular medications is discharge record; ‘N’: there is no discharge record but follow-up information with specified time if available. No information during hospital admission has been collected in our review. For example: ‘N, 1 mth’ is referring to the recorded prevalence of cardiovascular medications in 1 moth time after hospital admission.

The current review includes 28,000 MI or ACS patients, with patient numbers ranging from 110 [35] to 10753 [45] per study. The characteristics of participants also varied considerably. Sixteen out of 35 studies reported discharge medications after hospital admission [18,19,21–23,35,41,49] or had specified the time period after discharge [17,26,27,29,34,36,37,44], whereas others set no time limits. Further details on study characteristics are presented in S3 Table.

### Prevalence of cardiovascular medication use

**Fig 2:**
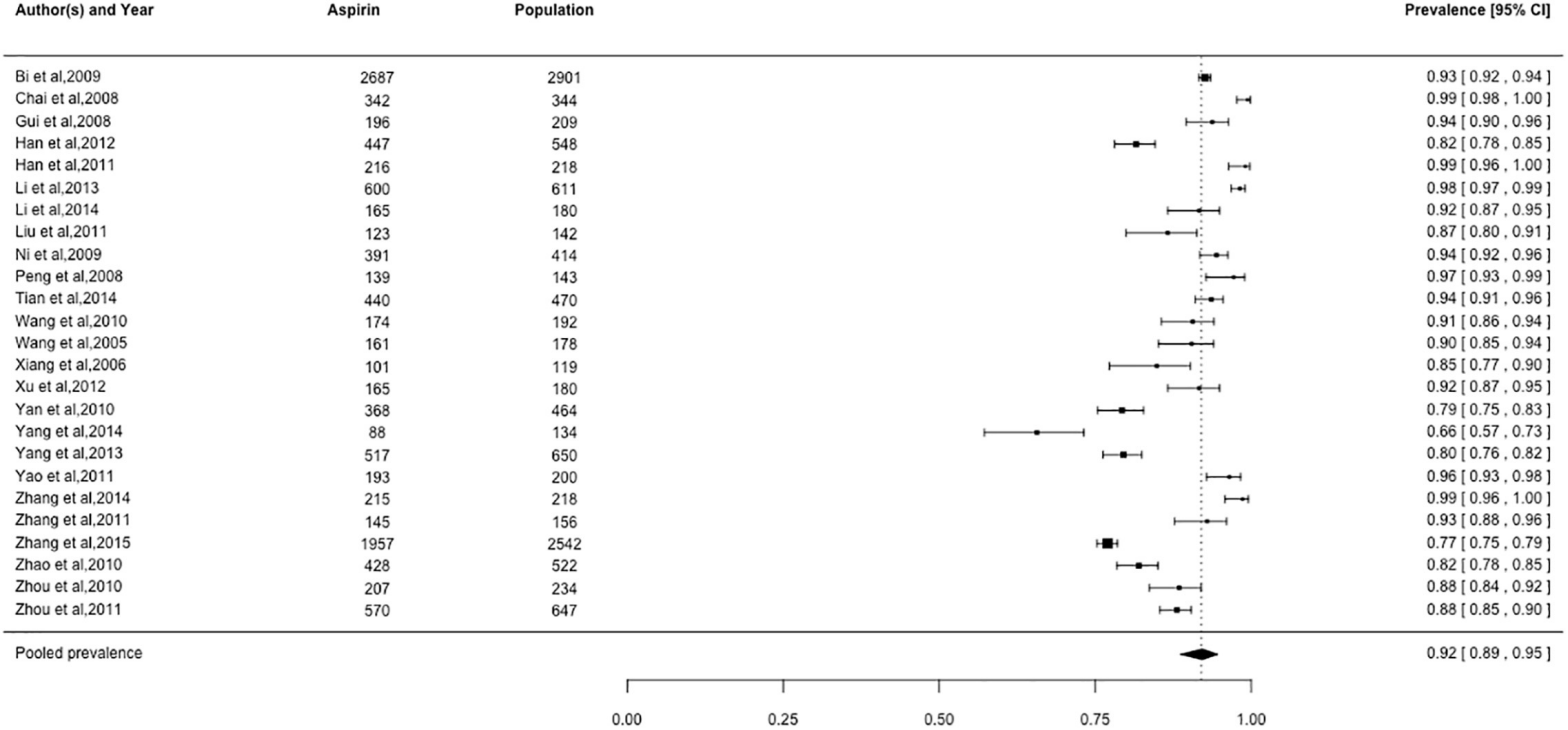
Prevalence of aspirin by Chinese myocardial infarction patients after hospital admission. Studies followed alphabetical order. Squares and the horizontal lines represent the measures of effect (odds ratio) and associated confidence intervals for each of the studies and the diamond indicates the summary measure.

**Fig 3:**
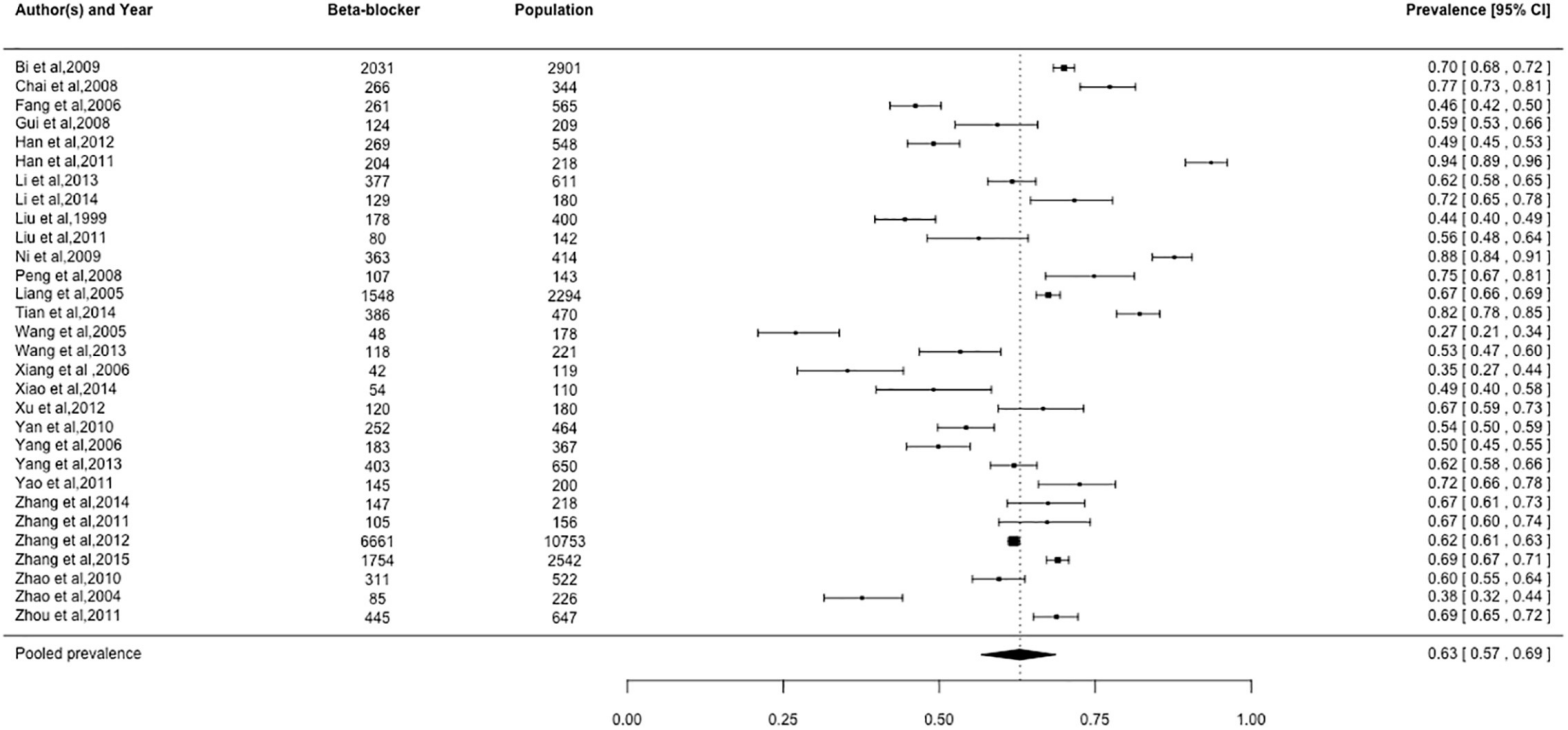
Prevalence of beta-blockers by Chinese myocardial infarction patients after hospital admission. Studies followed alphabetical order. Squares and the horizontal lines represent the measures of effect (odds ratio) and associated confidence intervals for each of the studies and the diamond indicates the summary measure.

**Fig 4:**
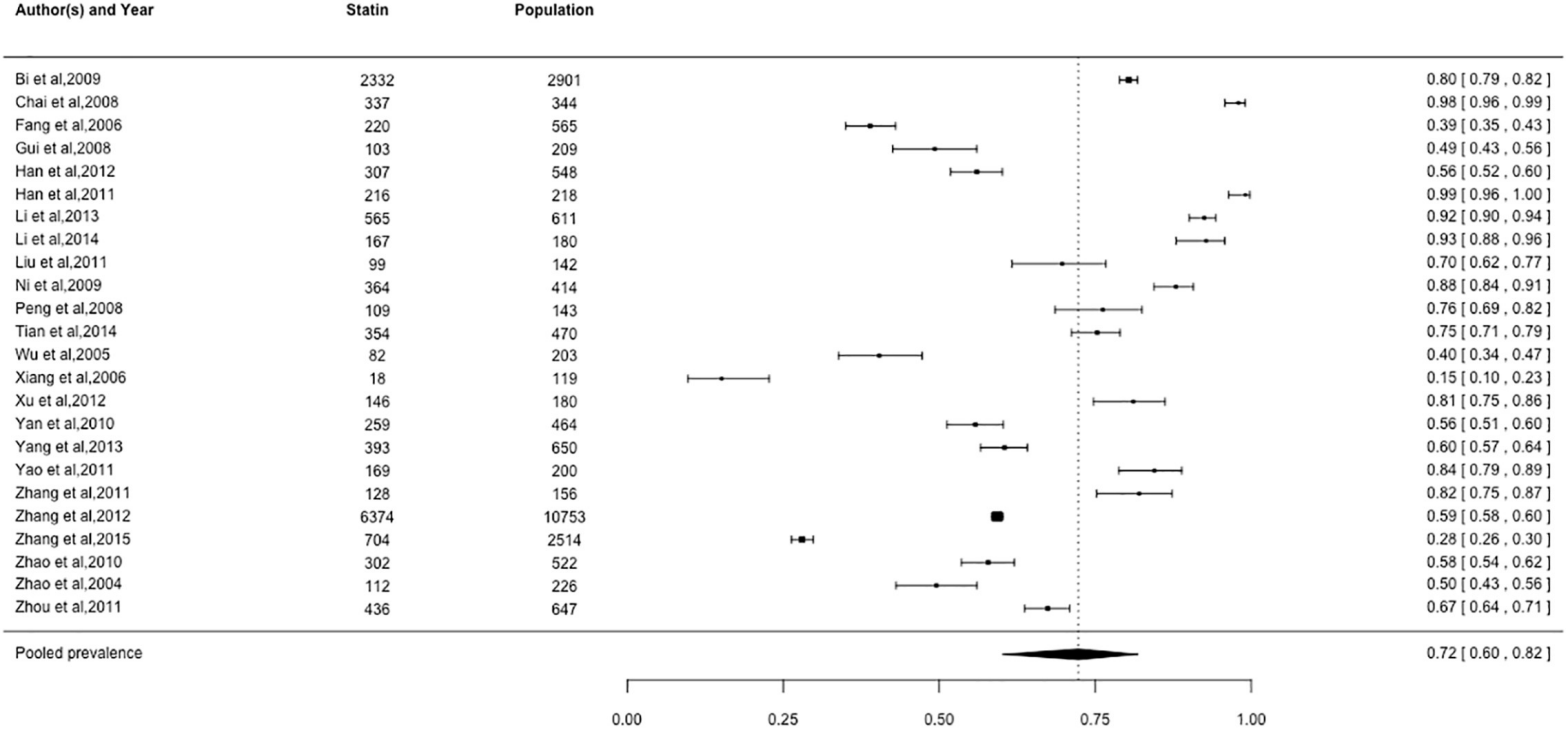
Prevalence of statins by Chinese myocardial infarction patients after hospital admission. Studies followed alphabetical order. Squares and the horizontal lines represent the measures of effect (odds ratio) and associated confidence intervals for each of the studies and the diamond indicates the summary measure.

**Fig 5:**
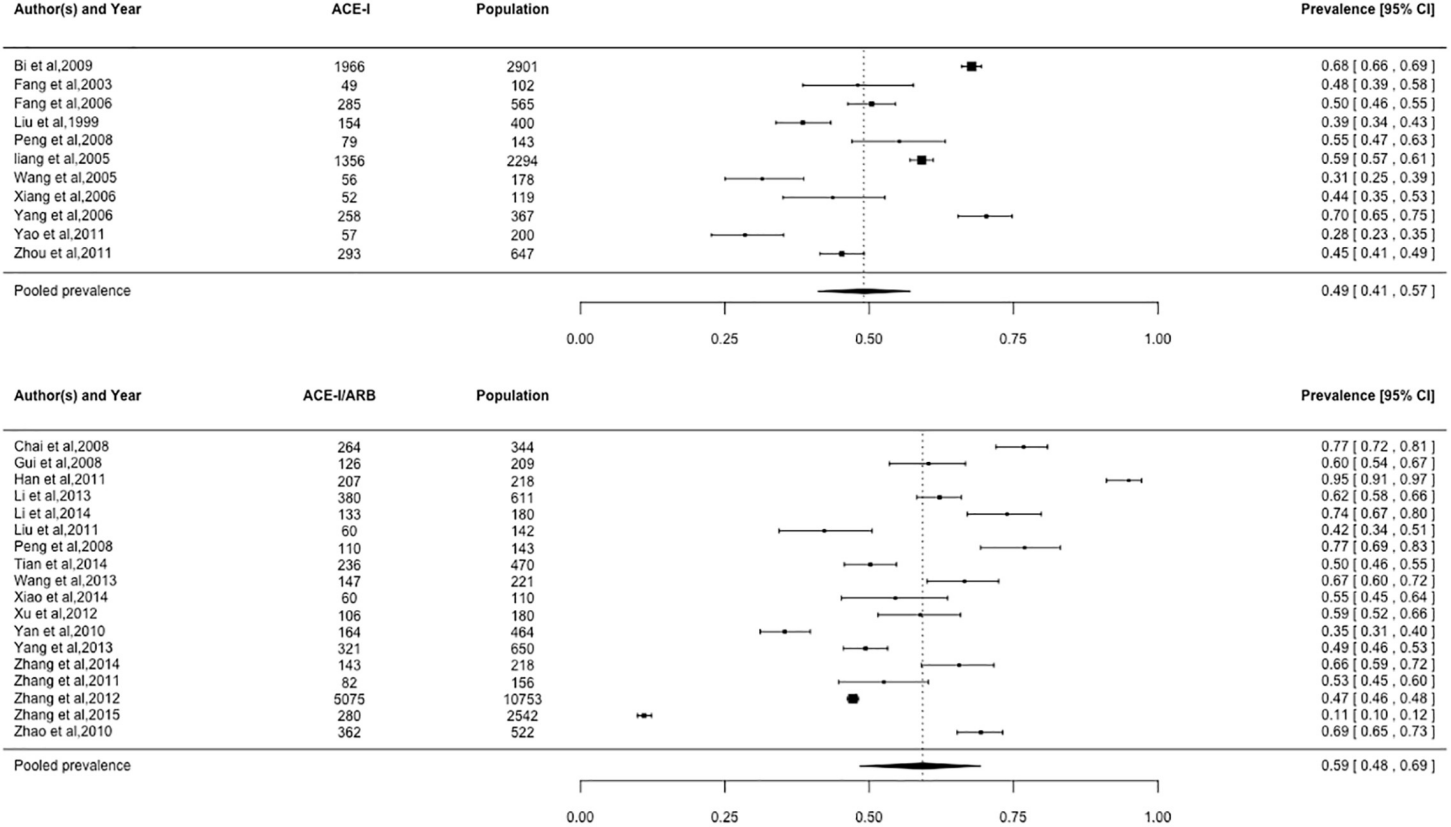
Prevalence of ACE-I/ARBs by Chinese myocardial infarction patients after hospital admission. Studies followed alphabetical order. Squares and the horizontal lines represent the measures of effect (odds ratio) and associated confidence intervals for each of the studies and the diamond indicates the summary measure. ACE-I: ACE-inhibitor; ARB: angiotensin receptor blocker.

**Fig 6:**
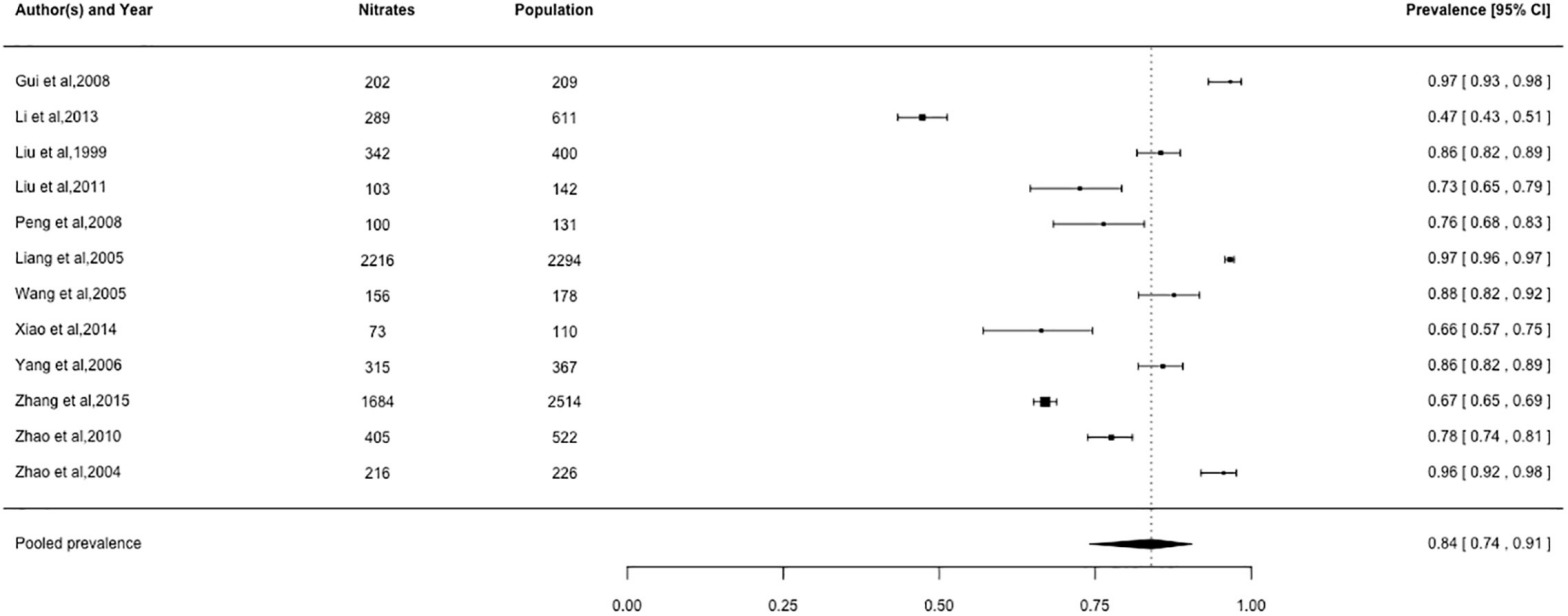
Prevalence of nitrates by Chinese myocardial infarction patients after hospital admission. Studies followed alphabetical order. Squares and the horizontal lines represent the measures of effect (odds ratio) and associated confidence intervals for each of the studies and the diamond indicates the summary measure.

Pooled prevalence estimates for cardiovascular medication use are presented in Figs 2–6, respectively. Among these six cardiovascular medication categories, the pooled prevalence rate was 92% for aspirin (95% CI: 0.89–0.95), 63% for beta-blockers (95% CI: 0.57–0.69), 72% for statins (95% CI: 0.60–0.82), 49% for ACE-Is (95% CI: 0.41–0.57), 59% for ACE-I/ARBs (95% CI: 0.48–0.69), and 84% for nitrates (95% CI: 0.74–0.91).

### Temporal trends in prevalence of cardiovascular medication use

**Fig 7:**
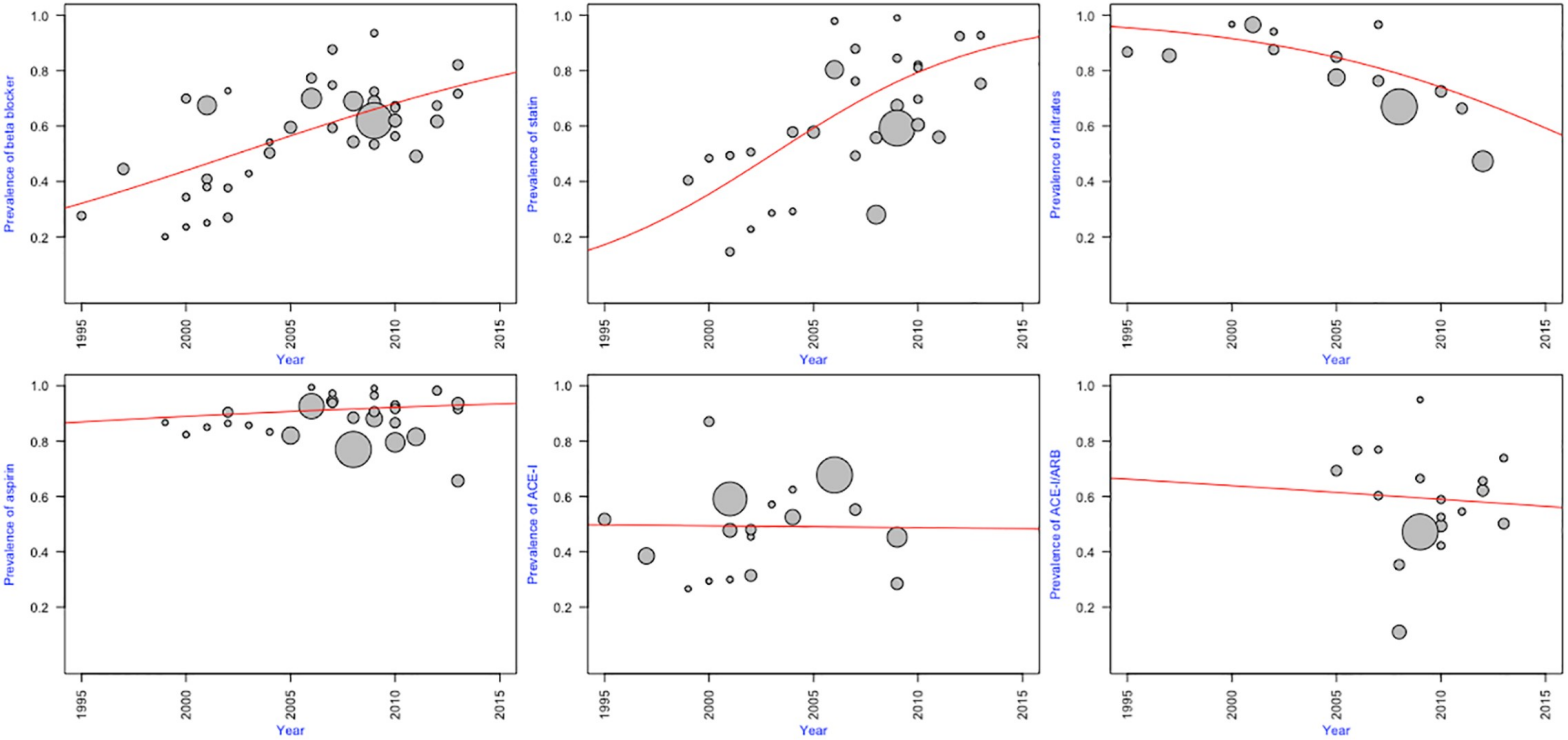
Temporal trends in the prevalence of cardiovascular medication use. Bubbles are individual studies; diameters of the bubbles are proportional to studies weight for analysis. ACE-I: ACE-inhibitor; ARB: angiotensin receptor blocker.

Fig 7 illustrates year-specific prevalence of individual cardiovascular medication use from 1995 to 2015. The meta-regression of the year over logit-transformed prevalence showed a trend towards an increasing prevalence of beta-blocker use from 1995 to 2015 with a slope of 0.1 (p<0.0001). This gives an estimate of 78% for beta-blockers use in 2015, compared to 32% in 1995. A similar increasing trend was demonstrated for statins use, even when the first available study was from 1999 (slope = 0.26; P = 0.0004). Accordingly, the estimated statins use was 17% in 1995 and 91% in 2015. In contrast, the estimated prevalence of nitrates use dropped from 95.5% in 1995 to 59.3% in 2015. There was no significant association between the year of survey and prevalence of aspirin, ACE-Is, and ACE-I/ARB use.

### Other demographic and geographic factors

Among studies that reported either demographic (the mean age and proportions of women) or geographic characteristics, there was little evidence for an association between these study characteristics and the logit-transformed prevalence of cardiovascular medications, except for aspirin (S4 Table). Aspirin use showed significant association with mean age (slope: 0.26, P = 0.02), indicating that elderly patients with previous MI are more likely to take aspirin for their medical conditions.

## Discussion

Cardiovascular medications are considerably underused for secondary in China, even though a pronounced increase in beta-blocker and statin use has been noticed over the period from 1995 to 2015.

In the current review, the reported prevalence of cardiovascular medications in China varied widely across studies. The largest variation on prevalence of these six cardiovascular medications was reported for statin use, ranging from low (14.6%) [16] to high (99.1%) [29], among included studies. The prevalence variations of other cardiovascular medication use are also notable in this review. The lowest prevalence for beta-blockers, ACE-Is, ACE-I/ARB, and nitrates was 27% [15], 31.5% [15], 35.3% [25], and 47.3% [32] in comparison to the highest prevalence with 93.6% [29], 67.8% [21], 95% [29], and 96.8%19 reported, respectively. Aspirin showed the least variation for reported prevalence, varying from 65.7% [36] to 99.4% [18].

To investigate the determinants of these variations in prevalence of cardiovascular medication use, meta-regression models were performed with specific study characteristics as covariates in current review. In line with previous findings [50,51], elderly patients with previous MI were observed to be more likely to take aspirin. Aspirin is not only used to prevent cardiovascular events but also applied for other medical conditions [52] and thus, older patients may be more likely to report use of aspirin. Although several studies have demonstrated differences in use of cardiovascular medications by age, sex, or geographic area [51,53–55], no other significant associations between demographic and geographic characteristics and cardiovascular medication use were observed in the current study. However, it should be noted that Chinese national guidelines recommend cardiovascular medications as part of secondary prevention strategy for all MI patients in daily practice irrespective of age, sex, or geographic area. [6]

The observed trends of cardiovascular medication use are likely to be related to recent changes of the healthcare system, insurance coverage, and published national guidelines in China. [1,6,56] After the Chinese government implemented its healthcare system reform policies in 2009 [1] and increased its insurance coverage up to 95.7% by 2011 [57], the availability of health-care access and affordability of medication prescription have improved considerably in China. [5,58] Introduction and regular updates of the Chinese national guidelines of MI were additionally used to complement this renewed healthcare system to standardize physician’s daily practice and improve quality of care. [6,10] Furthermore, the Chinese National Essential Medicine List (EML) was developed and is expected to be fully available by 2020 to support rational medication use and improve the access to safe and effective essential medication [5,21]. Already, a notable reduction of inappropriate drug prescriptions has been observed after EML implementation. [59] Reflecting these achievements, the use of guideline-recommended cardiovascular medications is expected to gradually increase in China.

Despite material healthcare improvements in China, it is also important to realize that the current review shows that cardiovascular medication is not on par with guideline recommendations indicating insufficient guideline implementation in daily practice. Studies have shown Chinese physicians to have a low awareness of up-to-date guidelines [60,61], affecting clinical decisions in spite of guideline recommendations [61–63]. Moreover, lack of knowledge among patients about their disease or the necessity of adequate treatment could also have contributed to low use of cardiovascular medications. [60] Notwithstanding improvements of the healthcare system and wider insurance coverage in China, CVD patients still face high personal expenditures on CVD care [64]. The Chinese national Bureau of Statistics showed private (out-of-pocket) expenditures to increase approximately by 10% per year [65], despite 20% annual increase on healthcare budget from government. Thus given high out-of-pocket expenditures, Chinese patients may face considerable financial hardship after a MI event, which may hamper their ability to manage their medical conditions, seek proper medical advice, or adhere to prescribed medications [56,66,67], calling an increased focus on the provision of accessible and to affordable healthcare service for the Chinese population.

Inadequate guideline-recommended cardiovascular medication use has been properly reported for other low- and middle- countries. [68,69] The PURE study, a large international observational study in 30 countries, indicated a low use of the antiplatelet, beta-blockers, ACE-I/ARB and statins in South Asia (11.6%, 11.9%, 6.4%, and 4.8%), Malaysia (14.9%, 12.5%, 12.8%, and 15.9%), and Africa (3.4%, 1.9%, 6.8%, and 1.4%) and demonstrated the challenges of affordability and availability of these medications. [67,68,70] Generally, these medications were observed to be more commonly available and affordable in high-income countries with more advanced healthcare systems and better quality of care in daily practice [67], resulting in higher reported cardiovascular medication use in these countries. [71,72] Unless both healthcare system and insurance coverage are improved towards wider availability and affordability of these medications, the use of guideline-recommended cardiovascular medication is likely to remain low in many low- and middle-income countries.

There are several limitations to this review. First, most published studies in China are more likely to come from centers with more advanced healthcare service and better facilities. In contrast, prevalence of cardiovascular medication is rarely reported and published in centers with limited resources, especially in rural areas. In the current review, comparative data collected from rural area is lacking and thus, due to limited availability of published data, we could not perform detailed stratified analysis of cardiovascular medication use by urban and rural area. Findings from these included, generally more urban, studies of our review may thus not be representative for the whole country. This is a source of bias potentially overestimating the prevalence of cardiovascular medication use in China. A representative study with a comprehensive investigation on the prevalence of cardiovascular medication in China is thus warranted. Secondly, this meta-analytic study collected its data from a large number of published observational studies with diverse study designs and population characteristics resulting in substantial study heterogeneity (S5 Table) and indicating potentially adverse effects on the stability and interpretability of the pooled prevalence estimates. Variability of the reported prevalence reflects cardiovascular medication use in daily practice. Application of random effect models and analyzed associated covariates explain these observed variations, although not individual patient level, limiting the potential of the current review to disclose associations between patient characteristics and cardiovascular medication use.

The strength of this review lies in its use of four databases to systematically search for articles including a large Chinese database. CNKI was used as a supplementary searching platform to incorporate both English and Chinese literature and to minimize limited access to Chinese publications from English language databases. Comprehensive Chinese searching terms were also used as part of our search strategy to cover literature published in China. Secondly, after validating all selected studies by a comprehensive quality assessment tool, meta-analysis and meta-regression models were performed to summarize the pooled estimates of cardiovascular medication use in China and assess its determinants. We observed significant year trends in prevalence of cardiovascular medication use for secondary prevention of MI over the last two decades, reflecting the rapid epidemiological changes in China. To our knowledge, this is the first review to investigate the trends of cardiovascular medication prevalence in China.

In summary, the current cardiovascular medication use in China is inadequate in comparison to guideline recommendations, although the reported use of these medications has increased over the last two decades. This should act as a wake-up call for policymakers and healthcare bodies to forcefully restructure and implement secondary prevention strategies, educate health professionals to update clinical knowledge and follow current guidelines recommendations for treatment, and create awareness among patients about health status and the benefits of appropriate medication.

## Supporting information

S1 Checklist PRISMA checklist. PRISMA checklist for ‘Prevalence of cardiovascular medication use on secondary prevention after myocardial infarction (MI) in China between 1995–2015: a systematic review and meta-analysis’.
(DOCX)

S1 List Study abbreviation list. (DOCX)

S1 Table Search strategy: search term and inclusion and exclusion criteria. (XLSX)

S2 Table Quality of risk bias assessment. Data collection and statistical analysis method were assessed in study design. Studies with summed score of 6 or below was considered as bad quality and excluded from this systematic review and meta-analysis.
(DOCX)

S3 Table Study characteristics. Numeric variables are number of measurements. Starting_yr: year when studies started data collection; Finishing_yr: year when studies finished data collection; mths: months. If medication information was collected from discharge letter, record it as ‘1’. If not, record it as ‘0’. Additionally, report recording time apart from discharge letter if available. For instance, 6 mths means information was collected 6 months after discharged.
(XLSX)

S4 Table Correlation of year, mean age proportion of women, and geographic area on prevalence of cardiovascular medications in China. Two decimals were applied. Significant P value was marked with asterisk (*).
(DOCX)

S5 Table Study heterogeneity of current systematic review and meta-analysis. ACE-I: ACE-inhibitor; Df: degree of freedom. All meta-analyses were applied with random-effects model.
(DOCX)
