# Supplementary material for: Prevalence of cardiovascular medication on secondary prevention after myocardial infarction in China between 1995-2015: A systematic review and meta-analysis
Source: PLoS One. 2017 Apr 20;12(4):e0175947. doi: 10.1371/journal.pone.0175947 (PMC5398555; doi:10.1371/journal.pone.0175947)
Supplement: S1 List — (DOCX) [file pone.0175947.s002.docx]

**S1 list. Study abbreviation list**

CVD Cardiovascular disease

MI Myocardial infarction

CENTRAL Cochrane Central Register of Controlled Trials

CNKI China National Knowledge Infrastructure

ACE-I ACE-inhibitor

ARB Angiotensin receptor blocker

BB Beta blocker

ACS Acute coronary syndromes

OR Odds ratio

CI Confidence interval

EML Essential medicine list
