# Supplementary material for: Prevalence of cardiovascular medication on secondary prevention after myocardial infarction in China between 1995-2015: A systematic review and meta-analysis
Source: PLoS One. 2017 Apr 20;12(4):e0175947. doi: 10.1371/journal.pone.0175947 (PMC5398555; doi:10.1371/journal.pone.0175947)
Supplement: S2 Table — Data collection and statistical analysis method were assessed in study design. Studies with summed score of 6 or below was considered as bad quality and excluded from this systematic review and meta-analysis. (DOCX) [file pone.0175947.s004.docx]

**S2 Table- Risk of bias**

**S2A Table: Adjusted tool of risk of bias assessment**

| **Bias type** | **Low risk** | **Moderate risk** | **High risk** |
| --- | --- | --- | --- |
| Study design | - Prospective data collection (clinical assessment) | - Retrospective data collection (medical records or self-reported questionnaire/survey) | - Unclear data collection and statistical analysis |
| Study population | - Specific and detailed sample selection criteria - Sample from general population but not selected group with multiple centres - Specific and detailed sample selection criteria | - Sample selected from large population but selection criteria not defined; - Sample selection ambiguous but may be representative; - Analysis to adjust for sampling strategy bias | - Unclear sample selection criteria; - Sample is selected from single centre and not representative |
| Participant rate | High participant rate (>85%) | Moderate participant rate (70-85%) | Low participant rate (<70%) |
| Participants’ characteristics | - Myocardial infarction (MI) diagnosis using consistent criteria and direct examination; - Specific and detailed recruitment time period; - Consecutive MI participants>18 years old; - Specific and detailed determinants (e.g. age) | - MI diagnosis assessment from medical records, questionnaire, survey, administrative database or register; - Wide and undetailed recruitment time period; - Specific and detailed determinants | - Diagnosis assessment from non-validated data or generic estimate from overall population; - Unknown performed time or location; - No determinants information available. |
| Outcomes | - Detailed information on prevalence of cardiovascular medications usage with specific MI diagnosis; - Detailed information on absolute level of blood pressure, lipids, and glucose with specific MI diagnosis | - Detailed information on prevalence of cardiovascular medications usage with specific MI diagnosis; | - No outcome information available |

**S2B Table: Quality of risk bias assessment**

| Study | Study design | Sample population | Participation rate | Participants' characteristics | Outcomes | Summed score |
| --- | --- | --- | --- | --- | --- | --- |
| Ni et al2009 | 1 | 1 | 2 | 1.5 | 1 | 6.5 |
| Liu et al 1999 | 1 | 2 | 0.5 | 2 | 1 | 6.5 |
| Liu et al 2011 | 1 | 0.5 | 2 | 0.5 | 0.5 | 4.5 |
| Liu et al 2001 | 1 | 1 | 0.5 | 2 | 2 | 6.5 |
| Liu et al 2005 | 1 | 0.5 | 2 | 0.5 | 0.5 | 4.5 |
| Liu et al 2010 | 1 | 1 | 2 | 1 | 1 | 6 |
| Bao et al 2013 | 1 | 1 | 2 | 1 | 0.5 | 5.5 |
| Xiang et al 2006 | 1 | 1 | 2 | 1 | 1 | 6 |
| Wu et al 2005 | 1 | 1 | 2 | 1 | 1 | 6 |
| Zhou et al 2010 | 2 | 1 | 2 | 2 | 2 | 9 |
| Yao et al 2011 | 2 | 1 | 2 | 1 | 1 | 7 |
| Sun et al 2014 | 1 | 0.5 | 2 | 1 | 1 | 5.5 |
| Ji et al 2004 | 1 | 0.5 | 2 | 0.5 | 1 | 5 |
| Zhang et al 2014 | 2 | 1 | 2 | 1.5 | 1 | 7.5 |
| Zhang et al 2009 | 1 | 0.5 | 2 | 1 | 0.5 | 5 |
| Zhang et al 2010 | 1 | 0.5 | 2 | 0.5 | 0.5 | 4.5 |
| Zhang et al 2011 | 2 | 1 | 2 | 2 | 2 | 9 |
| Zhang et al 2012 | 2 | 2 | 2 | 0.5 | 1 | 7.5 |
| Zhang et al 2005 | 1 | 1 | 2 | 2 | 1 | 7 |
| Peng et al 2008 | 1 | 1 | 2 | 2 | 2 | 8 |
| Fang et al 2003 | 1 | 1 | 1 | 2 | 1 | 6 |
| Fang et al 2001 | 1 | 1 | 2 | 1 | 1 | 6 |
| Fang et al 2006 | 1 | 1 | 2 | 0.5 | 1 | 5.5 |
| Fang et al 2006 | 1 | 1 | 2 | 1 | 2 | 7 |
| Li et al 2013 | 1 | 1.5 | 1 | 2 | 2 | 7.5 |
| Li et al 2014 | 1 | 1 | 2 | 2 | 2 | 8 |
| Yang et al 2014 | 1 | 1 | 2 | 1 | 2 | 7 |
| Yang et al 2006 | 1 | 1 | 2 | 2 | 1 | 7 |
| Chai et al 2008 | 1 | 1 | 2 | 1 | 2 | 7 |
| Gui et al 2008 | 1 | 1 | 2 | 1 | 1 | 6 |
| Wang et al 2010 | 1 | 1 | 2 | 2 | 1 | 7 |
| Wang et al 2005 | 1 | 1 | 2 | 1 | 1 | 6 |
| Wang et al 2013 | 2 | 2 | 2 | 1 | 1 | 8 |
| Tian et al 2014 | 1 | 1 | 2 | 1.5 | 2 | 7.5 |
| Niu et al 2003 | 1 | 1 | 2 | 0.5 | 1 | 5.5 |
| Luo et al 2014 | 1 | 1.5 | 2 | 0 | 1 | 5.5 |
| Xiao et al 2014 | 1 | 1 | 2 | 1 | 2 | 7 |
| Xiao et al 2012 | 1 | 0.5 | 0.5 | 0.5 | 0.5 | 3 |
| Xu et al 2012 | 1 | 1 | 1 | 1.5 | 2 | 6.5 |
| Xie et al 2012 | 1 | 0.5 | 2 | 1 | 1 | 5.5 |
| Tan et al 2013 | 1 | 0.5 | 2 | 1 | 1 | 5.5 |
| Zhao et al 2010 | 1 | 1 | 2 | 2 | 2 | 8 |
| Zhao et al 2004 | 1 | 1 | 2 | 1 | 2 | 7 |
| Lang et al 2006 | 1 | 1 | 2 | 1 | 2 | 7 |
| Guo et al 2012 | 1 | 0.5 | 2 | 1 | 1 | 5.5 |
| Tao et al 2014 | 1 | 0.5 | 2 | 1 | 1 | 5.5 |
| Han et al 2012 | 1 | 1 | 2 | 1 | 2 | 7 |
| Han et al 2011 | 1 | 1 | 2 | 1 | 2 | 7 |
| Gao et al 2007 | 1 | 0.5 | 2 | 1 | 1 | 5.5 |
| Bi et al 2009 | 2 | 2 | 2 | 2 | 1 | 9 |
| Liang et al 2005 | 2 | 2 | 2 | 1 | 1 | 8 |
| Ma et al 2010 | 2 | 1 | 1 | 0.5 | 0.5 | 5 |
| Wang et al 2012 | 2 | 0.5 | 0 | 1 | 1 | 4.5 |
| Yan et al 2010 | 2 | 0.5 | 2 | 1 | 1 | 6.5 |
| Zhang et al 2015 | 2 | 1 | 2 | 1 | 1 | 7 |

* Data collection and statistical analysis method were assessed in study design. Studies with summed score 6 or below was considered as bad quality and excluded.
