# Supplementary material for: Prevalence of cardiovascular medication on secondary prevention after myocardial infarction in China between 1995-2015: A systematic review and meta-analysis
Source: PLoS One. 2017 Apr 20;12(4):e0175947. doi: 10.1371/journal.pone.0175947 (PMC5398555; doi:10.1371/journal.pone.0175947)
Supplement: S4 Table — Two decimals were applied. Significant P value was marked with asterisk (*). (DOCX) [file pone.0175947.s006.docx]

**S4 Table**

**S4A Table Correlation of year, mean age, and proportion of women on prevalence of cardiovascular medications in China**

ACEI: ACE-inhibitor. Two decimals were applied. Significant P value was marked with asterisk (*)

**S4B Correlation of geographic area on prevalence of cardiovascular medications in China**

* NA: not applicable; ACEI: ACE-inhibitor. Two decimals were applied. Significant P value was marked with asterisk (*).
