# Supplementary material for: Prevalence of cardiovascular medication on secondary prevention after myocardial infarction in China between 1995-2015: A systematic review and meta-analysis
Source: PLoS One. 2017 Apr 20;12(4):e0175947. doi: 10.1371/journal.pone.0175947 (PMC5398555; doi:10.1371/journal.pone.0175947)
Supplement: S5 Table — ACE-I: ACE-inhibitor; Df: degree of freedom. All meta-analyses were applied with random-effects model. (DOCX) [file pone.0175947.s007.docx]

**S5 Study heterogeneity of current systematic review and meta-analysis**

|  | I^2^ Statistics | Q Statistics | Df | P value |
| --- | --- | --- | --- | --- |
| Aspirin | 99.0% | 676.7 | 25 | <0.1% |
| Beta blocker | 98.8% | 814.4 | 29 | <0.1% |
| Statin | 99.7% | 2290.6 | 24 | <0.1% |
| ACE-inhibitor | 97.5% | 373.6 | 10 | <0.1% |
| ACE-I/ARB | 99.1% | 1532.3 | 17 | <0.1% |
| Nitrates | 99.4% | 900.8 | 12 | <0.1% |

ACE-I: ACE-inhibitor; Df: degree of freedom. All meta-analyses were applied with random-effects model
